# Volume Fabrication of Quantum Cascade Lasers on 200 mm-CMOS pilot line

**DOI:** 10.1038/s41598-020-63106-4

**Published:** 2020-04-10

**Authors:** J. G Coutard, M. Brun, M. Fournier, O. Lartigue, F. Fedeli, G. Maisons, J. M Fedeli, S. Nicoletti, M. Carras, L. Duraffourg

**Affiliations:** 1 Univ. Grenoble Alpes, CEA, LETI, F38054 Grenoble, France; 2 mirSense - Centre d’intégration NanoINNOV, Bâtiment 863, 8 avenue de la Vauve, F91120 Palaiseau, France

**Keywords:** Nanoscience and technology, Optics and photonics

## Abstract

The manufacturing cost of quantum cascade lasers is still a major bottleneck for the adoption of this technology for chemical sensing. The integration of Mid-Infrared sources on Si substrate based on CMOS technology paves the way for high-volume low-cost fabrication. Furthermore, the use of Si-based fabrication platform opens the way to the co-integration of QCL Mid-InfraRed sources with SiGe-based waveguides, enabling realization of optical sensors fully integrated on planar substrate. We report here the fabrication and the characterization of DFB-QCL sources using top metal grating approach working at 7.4 µm fully implemented on our 200 mm CMOS pilot line. These QCL featured threshold current density of 2.5 kA/cm² and a linewidth of 0.16 cm^−1^ with a high fabrication yield. This approach paves the way toward a Mid-InfraRed spectrometer at the silicon chip level.

## Introduction

These two last decades, hybrid photonic circuits using both silicon and III-V materials have been successfully developed for data communication and are a complementary technology to advanced CMOS for high performance computers too. In 2006, Soref suggested to consider a similar technological approach to make photonics circuits dedicated to the mid/long wave infrared region^1^. This IR-wavelength range enables to address multiple applications from free-space data communication^2^, optical countermeasures^3^, IR imaging of biological tissues^4^ to spectroscopy^5^. So far, the detection of chemical species in solid^6^, liquid^7^ or gas mixtures^8^ remains the most popular application that drives the technological development. In fact, the spectral range between 3 μm and 12 μm corresponds to the first harmonic resonance between rovibrational energy levels of most of chemical species, leading to absorption cross sections that are order of magnitude stronger than overtone in the near IR for instance. This is especially true for light molecules in gaseous phase. This unique feature enables to detect a great number of gases with high sensitivity and selectivity. A limit of detection lower than 1 part per billion (ppb) and unequivocal identification of chemical species can typically be reached through Mid-InfraRed (MIR) absorption spectroscopy techniques^9^.

Chemical detection with laser diodes has been widely developed since mid-1960s. Nevertheless, the emergence of unipolar sources based on multiple quantum well stacks has transitioned spectroscopic sensing in the MIR wavelength band toward portable systems useful for industrial applications. The technological progresses done on Quantum Cascade Laser (QCL)^10,11^, and Interband Cascade Laser (ICL) make these MIR sources compact and reliable and now readily available^12^. Distributed FeedBack (DFB) lasers are of particular interest as they provide a narrow and specific emission spectrum useful for the detection of chemicals^13,14^. In particular, they offer new detection solutions of gases in real time, *in situ* and at ultra-low concentration level.

With MIR Si-based photonic solutions, a novel class of integrated components has been developed allowing the integration at chip level of the main building blocks required for chemical sensing, i.e. the source, photonic integrated circuits (PICs) and the detector^15^. At wavelengths around 4.8 µm, Spott *et al*. developed a silicon on SiN waveguide coupled with a bonded QCL material. DFB lasers with threshold currents as low as 80 mA and threshold current densities below 1 kA/cm2 emitted more than 200 mW from a polished III-V/Si facet, and operated in pulsed mode up to 100 °C^16^. They rely on InP type fabrication using III/V-manufacturing lines on two inches format.

Such technological approaches cannot satisfy volume markets. Today manufacturing price may be estimated around 1k€ per laser after fabrication, test, sorting and bonding of the die on its holder. To date, even if III-V technologies are sufficient to meet the needs of niche markets they cannot tackle the QCL volume manufacturing challenges: implementation of automatic testing procedures at the wafer level, improvement of the reproducibility of electro-optical features, simplification of the sorting operations and implementation of a quality-control system.

So far, these challenges have not been properly addressed and prevent from a large development and dissemination of these lasers beyond scientific community and niche applications. In this paper, we present an original approach for the MIR based on the use of microelectronic tools to realize the fabrication of QCL on 200 mm Si wafers. Doing so, we are able to dramatically increase the laser reproducibility, while preserving the same performances as those reached on InP and to set-up automatic test procedure for reducing or even removing the sorting of lasers. This paves the way to the manufacturability of low cost devices suitable for numerous applications ranging from single laser to complete analysis system of chemicals, biologicals at liquid or gas phases. This work addresses a fundamental building block for the co-integration of QCL array with suitable MIR PICs and PhotoAcoustic detectors for making a spectrometer fully miniaturized at chip level.

Today, the silicon QCL is butt-coupled with our photoacoustic cell with a current pick and place tool. We are developing evanescent coupling between the QCL and MIR PICs placed underneath too. In parallel MIR waveguides have been manufactured within our silicon photoacoustic cell to have a fully integrated photoacoustic spectroscopic system.

**Figure 1:**
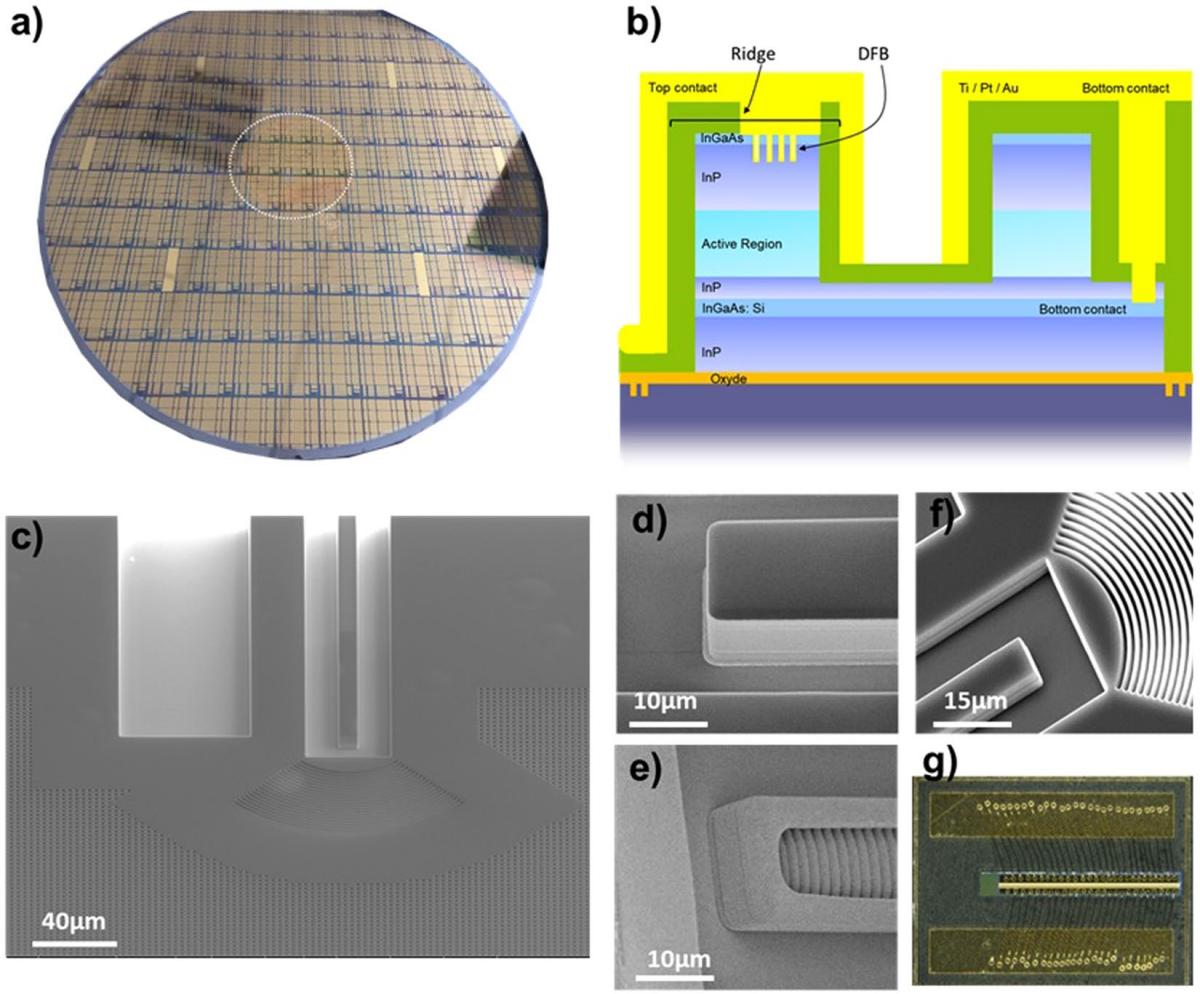
(**a**) 200 mm Si wafer with QCL components (the dotted circle refers to plate 2″ InP) – (**b**) Schematic view of the laser ridge at the end of the fabrication – (**c**) SEM top view of the laser ridge: the dark part corresponds to the output without metal, the light part is the metallized DFB – (**d**) Zoom in of the laser output – (**e**) Back mirror & metallized DFB – (**f**) Zoom in of the grating used for collective measurements – (**g**) Single laser mounted on AlN support.

This paper reports on the design, fabrication, and characterization of DFB-QCL single sources and arrays made on 200 mm Si wafers (Fig. 1a)) and compares their performances with QCLs made on InP substrate, with designs similar to those used in this work. For the sake of clarity, we will mainly focus on the devices emitting at 7.4 µm, even if similar experimental results have been obtained at 4.5 µm.

## Design

QCLs are commonly based on III-V materials depending on the chosen wavelength range. Most of the QCL emitting in the MIR region (in particular between 3 µm and 11 µm) are made from a stack of InGaAs/AlInAs layers on InP substrate. These heterostructures are significantly more efficient than the GaAs/AlGaAs stacks and reach optical power up to few watts^17,18^, in continuous mode and at room temperature. InAs based QCL are also good candidates in particular for short wavelengths around 3 µm. More recently, Baranov and co-workers have demonstrated InAs/AlSb QCL emitting at 15 µm wavelength with a low threshold current density at room temperature^19^.

The QCL developed within this work will be used for chemical analysis in the 4–10 µm wavelength range. The design has been derived from previous stacks developed a couple of years ago and specifically designed to operate in the 7.4 µm emission wavelength band. The laser heterostructure is a standard lattice-matched GaInAs and AlInAs multilayers grown on InP. Our active region is composed of 25 elementary periods, which are composed of a 3-quantum-wells and a superlattice resonant injection regions. For more details on the heterostructure, the reader can refer to^13^.

The electroluminescence spectrum of the epitaxy layers, measured at 80 K, showed that the emission band is actually centered at 1400 cm^−1^ (7.1 µm) with a full width at half maximum, *FWHM* = 14.4 *meV*. The multi-layer stack designs were however modified from DFB-QCL sources originally done on InP to take into account the constraints set by CMOS processing tools. The bottom contacts of the lasers are in particular no longer on the backside of the chips, but reported on one side of the laser introducing doped layers below the active region.

Three lengths of ridge have been investigated (*L*_*ridge*_ = 1,2, and 4 *mm*). We designed four ridge widths for each length (*w*_*ridge*_ = 6,8,10 and 12 *μm*) to study the current density threshold and the related overlap with the active region that should degrade the output power at same injected current density level. The nominal ridge geometry is 10 µm-wide, 2 mm-long. It corresponds to a good compromise between gain and losses and enables to have a single spatial mode. This width is adjusted to increase the losses of the higher order modes to the benefit of the first spatial mode. The DFB-QCL requires a wavelength selection mechanism, done with a metal surface grating according to the approach coupling a surface plasmon-polariton mode and the propagative modes^13^.

## Technology and fabrication

The implementation of QCL fabrication process on silicon benefits from the specific DFB technology developed a few years ago^16,20^, and routinely implemented on InP^13,21^.

The manufacturing of DFB QCL is realized through a grating in the III-V stack having a periodic index variation. This approach implies regrowth of III-V material after the grating etching^10^. Today, this regrowth is not possible in a CMOS-like manufacturing of QCL on silicon wafer. To overcome this issue, the wavelength selection, presented in^16^, is achieved on a silicon/nitride waveguide. This approach requires coupling the QCL cavity to a silicon/oxide waveguide, which has two main drawbacks. It complicates the design to have an efficient coupling inside the laser cavity, and reduces the useful wavelength range due to the presence of oxides in the waveguide. Oxide usually exhibits high losses above 4 µm MIR wavelength (likewise SiN above 7 µm). The top metal grating approach in double trenches does not require regrowth and has therefore provided the basis for the manufacturing process that we present in this paper.

In this work, we manufactured five wafers, three centered on 7.4 µm emission wavelength and two centered on 4.5 µm emission wavelength. Typical 200 mm silicon wafers with the active 2″ area including the QCLs and a schematic of the laser cross section are shown in Fig. 1a,b) respectively. SEM images of the laser inputs and outputs are also presented on Fig. 1c–f). We stress that Fig. 1f) corresponds to the 2D grating that helps to decouple the light during the automatic measurement. This part is removed when dicing the wafer for chip level characterizations. The Fig. 1g) corresponds to one laser bonded on an aluminum nitride holder.

The performance of lasers depends not only on the quantum well stack but also on the morphology of the laser ridge and the DFB filter. SEM characterizations were performed to verify the geometry of the ribbon and the quality of its edges. The expected widths were larger by 1.4 µm. The manufacturing accuracy is in the order of +/−100nm over the entire center of the 200 mm wafer (Fig. 1a)). The roughness of the etching flanks is less than 100 nm (RMS) (see for example Fig. 1d)). The ribbon edges are metallized with gold to increase the losses of higher order optical modes and to have a single mode laser. It should be noted that the metallization of the back mirror allows a 95% reflectivity and that an anti-reflective layer (SiN) ensures a 95% transmission on the output facet. Hereafter we describe the five fundamental bricks of the fabrication process, namely: (i) InP/silicon wafer bonding, (ii) DFB definition, (iii) ridge etching, (iv) bottom and top contact fabrication and (v) metallization.

First, we directly bonded a 2 inch InP wafer with the active layers on a 200 mm Si wafer (Fig. 1a)) via a thin oxide layer. This process step is now a mature technology but it requires high-quality defect-free epilayers. This key process step has been addressed first by the study of the impact of the surface quality of the InP wafers with epitaxial quantum stack from different providers. The bonding is very sensitive to defects and requires a low roughness and a low bow. If the availability of defect-free epitaxial layers are primordial, the quality of the InP substrates is also critical. Providing that the surface defects are low enough on the epitaxial wafers, we achieved direct bonding of more than 90% of surface on oxidized 200 mm Si wafer. The InP substrate was first grinded and then fully removed by chemical etching of the different sacrificial layers in order to leave a perfect upper surface for further processing. We also estimated the layer uniformity across the 2″ area by transmission electron microscopy and profilometer. The relative dispersion of the stack thickness (active zone and cladding) across the 2″ area is around 0.7%. This value shows that the process is mastered and enables to perfectly control the final electrical features of the lasers. It is worth mentioning that a single Si wafer can receive up to four 2 inch wafers so that the number of devices manufactured on a single wafer can be increased from 2700 up to 10000 single lasers per wafer. This QCL manufacturing is fully compatible with standard microelectronics processes as it directly exploits the top metal grating technology, which does not require any further process on the InP wafer before the molecular bonding, ensuring the high volume rate.

The main challenge encountered in the fabrication of QCL within a 200 mm CMOS compatible facility was the development of suitable processes matching the etching of thick layers up to 10 µm and high resolutions down to 300 nm. The DFB was therefore realized immediately after the wafer bonding to perform a deep UV lithography at 248 nm on flat surface. The DFB was dry etched into the top InGaAs and InP layers through a first SiN hard mask with a typical pitch of 500 nm for the 7.4 µm wavelength.

The laser ridge was then defined by dry etching of the III-V layer stack up to the InP stop layer (see Fig. 1b)). This etching step was carried out through a second SiN hard mask. We realized the passivation of the laser ridge by a 1 µm thick conformal layer. It served as the electrical insulation layer as well as the anti-reflective layer. The bottom contact was performed by wet etching of the InP bottom layer with a stop on the InGaAs layer. Before metallization, the SiN was carefully removed from the DFB for having clean DFB patterns without any residues of SiN. The SiN layer was however preserved on the four sides, and used as the AR-coating for the output interface, see Fig. 1d). Thick conformal metallic layers (Ti/Pt/Au) were deposited both for the proper functioning of the DFB and for getting good contacts, Fig. 1e). We removed the metal layers with two successive wet and dry etch steps. The mask allowed us to preserve the metallic coating on the DFB and the rear mirror, Fig. 1e).

## Electro optic characterizations

As aforementioned, the fabrication of QCLs on 200 mm substrate enables to use large-scale characterization tools. Thus, 2700 devices (corresponding to single QCLs and QCL arrays) were measured at wafer-level on an automatic 200/300 mm test prober to determine the emission threshold levels. To do so, *V*(*I*) and *P*(*I*) characteristics were systematically measured using synchronized IR detector (VIGO system, Poland) to measure the output light emission. To capture the light emission from lasers, the angle between the wafer and the IR-detector was set at 51°, i.e. the decoupling angle of the grating shown in Fig. 1f). The decoupling efficiency around 0.5% was high enough to detect the threshold currents. With this set-up, we were able to measure the threshold power with a 2 × 10^−7^ W precision level corresponding to a 100 nA uncertainty over the threshold current. The measurements were performed in pulsed mode at low duty cycle (3%) and at 17 °C to avoid any self-heating effect. The pressure exerted on the electrical contact pads as well as the positioning of the prober tips remain delicate operations and may modify the serie resistances (typically in the order of 1Ω or less). Calibration of our prober has shown an average contact resistance of a few tens of mΩ (and a dispersion in the order of 1 mΩ), which is negligible compared to the differential resistance of our QCLs that are typically around 40Ω. We extracted the repartition of threshold current densities, *J*_*th*_, per wafer, which enables the selection of functional dies and the evaluation of the fabrication yield. In particular, we computed the average threshold currents and the standard deviations for each geometry of ridges.

**Figure 2:**
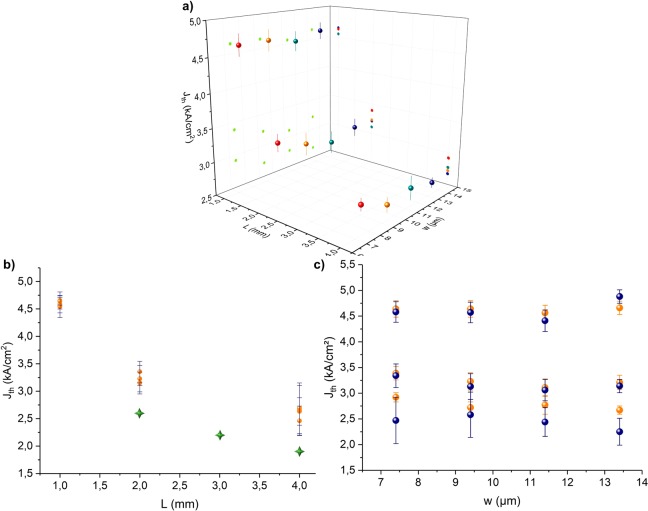
Current density thresholds: (**a**) average and standard deviation values with the length and the width (225 dies per geometry) for one typical 200 mm-wafer at 7.4 µm emission – (**b**) average and standard deviation values with width (orange circles: wafer 1, blue circles: wafer 2) – (**c**) average and standard deviation values as a function of length for the two silicon wafers (orange circles) and for lasers made on InP (green stars).

**Figure 3:**
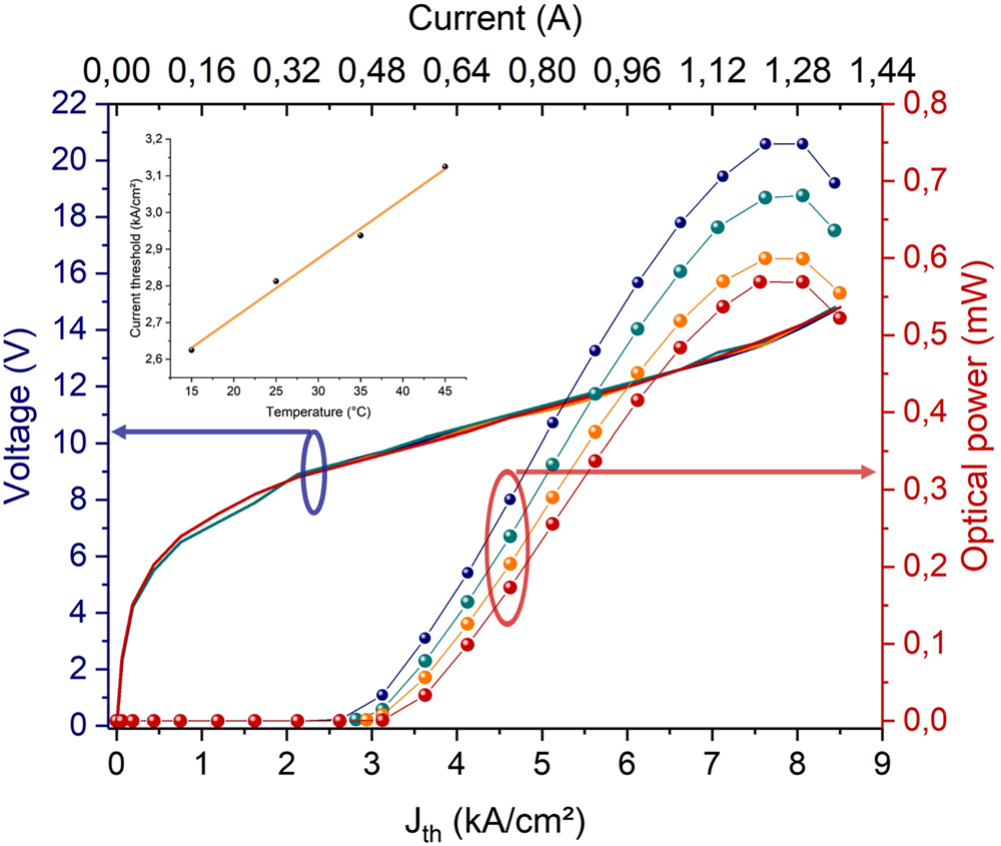
Typical characteristics *P*(*I*),*V*(*I*) of QCL lasers at 7.4 µm emission wavelength (ridge width=8 µm, ridge length=2 mm) at four temperatures: blue: 15 °C, green: 25 °C, orange: 35 °C and red: 45 °C – inset: *J*_*th*_(*T*) vs. temperature.

In Fig. 2, we have represented the threshold current density as a function of the length and width of lasers. Each point corresponds to the average value of the current density and its standard deviation computed over 225 identical lasers. Figure 2a) corresponds to measurements made on one typical wafer. Figure 2b) shows the averages and standard deviations computed from the cumulative measurements done on two wafers according to the ridge length. For reference, we have superimposed the typical (non-statistical) threshold current densities measured on lasers manufactured on InP. The active quantum wells of lasers on InP and those of lasers on silicon are the same. The top layers are the same too. The EPI layers have been grown on the same InP substrate and have the same quality. For the shortest lasers (1 mm), the threshold current density is around 4.6 kA/cm² and drops for the longest lasers (4 mm) down to 2.5 kA/cm². The threshold current decreases rapidly with the length of the DFB grating, whose reflectivity tends towards 1 from a value close to or greater than 4 mm. The losses of the laser ribbon become predominant from this length. The threshold current densities exhibited by QCL fabricated on InP substrate remain lower (2.6 kA/cm² & 1.9 kA/cm² for 2 mm and 4 mm respectively). This difference is probably due to the bottom layers we used for the bottom electrical contact. For InP device, the contact is usually made through the doped InP substrate and for Si the contact is made through a specific InGaAs:Si layer (see Fig. 1b). A thick InP layer on top of the bonding interface is for an optical confinement. This structural difference explains the difference in *J*_*th*_ between InP and Si. First, electrical simulations have shown that the electrical field lines are not perfectly vertical compared to two parallel electrode architecture like InP lasers. The current injection is then a bit less efficient even if the serie resistances remain comparable to those of InP devices. For optical confinement purpose we added thick InP layer (see Fig. 1b) – 3µm-thick). We carried out optical simulations to estimate the losses due to optical leakage through silicon. It turns out that there is no leakage through the silicon substrate. On the other hand, there are additional losses due to the contact layer InGaAs:Si close to the active area. But a formal correlation between the threshold degradation and these simulations have not been properly achieved yet.

Figure 2c) shows the average values and standard deviations as a function of width (values from SEM observations). We reported two sets of measurements corresponding to two different wafers. The orange circles are for the typical wafer (same as in Fig. 2a)) since the blue ones are for a second wafer from the same fabrication batch. There is no clear trend in the variation of the threshold current with the width. Nevertheless, for lengths of 1 mm and 2 mm, it seems that lasers around 10 µm wide have the lowest current densities. This observation is consistent with the initial sizing of the laser, whose theoretical optimal width was set at 10 µm for the stack considered. The values for the two wafers remain very close, in particular for 1 mm and 2 mm long lasers. The 4 mm long lasers of the second wafer show a larger dispersion in the measured current densities that is not well understood yet.

The relative standard deviation ${\sigma }_{{J}_{th}}/\overline{{J}_{th}}$ is about 3% except for the 4 mm long lasers of the second wafer. This weak dispersion demonstrates the good robustness of our technology. To go further, it is interesting to compare the disparity of the threshold current values with the dimensional dispersion related to our lithography/etching processes, used to structure the laser ribbon and DFB grating. The tolerance of the manufacturing process is better than 100 nm over a ridge width and does not induce the relative dispersion of few percent observed in Fig. 2. Moreover, the DFB design uses a coupling of the surface plasmon mode at the metal/dielectric interface with the guided mode^13,20^. This approach enables a coupling strength of the grating and losses that are almost constant over a wide range of etching depths^20^. We estimated that the acceptable tolerance depth is +/−100nm. The variation of our etching process remains below this limit (typically+/−50nm measured on few samples) what should not significantly influence the threshold current.

With this systematic electro-optical characterization, we estimated a yield of 98% of functional lasers per wafer (with similar electro-optical features).

After these first characterizations, the wafers were diced into discrete components (2700 dies/wafer), Fig. 1g). *P*(*I*) and *V*(*I*) were once again measured according to the applied current through the laser to extract *J*_*th*_ and the maximum current density *J*_*max*_. These measurements were performed at four different operating temperatures (from 15 °C to 45 °C) at 1.5%-duty cycle. When the current density reaches *J*_*max*_, the injection band level becomes higher than the transition level and the Stark rollover effect appears. In this regime, the optical power drops with the current. From the curve at different temperature, we estimated the variation of *J*_*th*_(*T*) and extracted the characteristic temperature *T*_0_ according to (1).

1 $${J}_{th}(T)={J}_{0}{e}^{\frac{T}{{T}_{0}}}$$

In the Fig. 3, we present typical *P*(*I*) and *V*(*I*)curves obtained for the nominal geometry (theoretical ridge width= 8 µm, measured width = 10.4 µm, ridge length = 2 mm). As shown in inset of Fig. 3, *T*_0_ is close to 176 K, which corresponds to quite common value obtained on InP substrate. This demonstrates that both the bonding process of quantum well stacks on Si wafers and the ridge /DFB structuration using CMOS compatible tools do not degrade the electro-optical performances of the QCL. The roll-off currents are quite common too. These characterizations have however been done at low duty cycle preventing from large self-heating occurrences and no systematic measurements of the heat diffusion through the Si substrate has been made so far. Further measurements at the die level are in progress to better characterize the QCL when working in pulsed mode up to 15%-duty cycle. In this case, the average optical powers would be between 5 mW and 10 mW.

A differential optical peak power $dP/dI$of ~83 mW/A can be easily estimated from measurements presented in Fig. 3 considering the duty cycle of 1.5%. For comparison, typical differential powers that can be found with commercial lasers on InP are around few 100 mW/A in similar conditions (in pulse mode and at 15°C)^22^.

**Figure 4:**
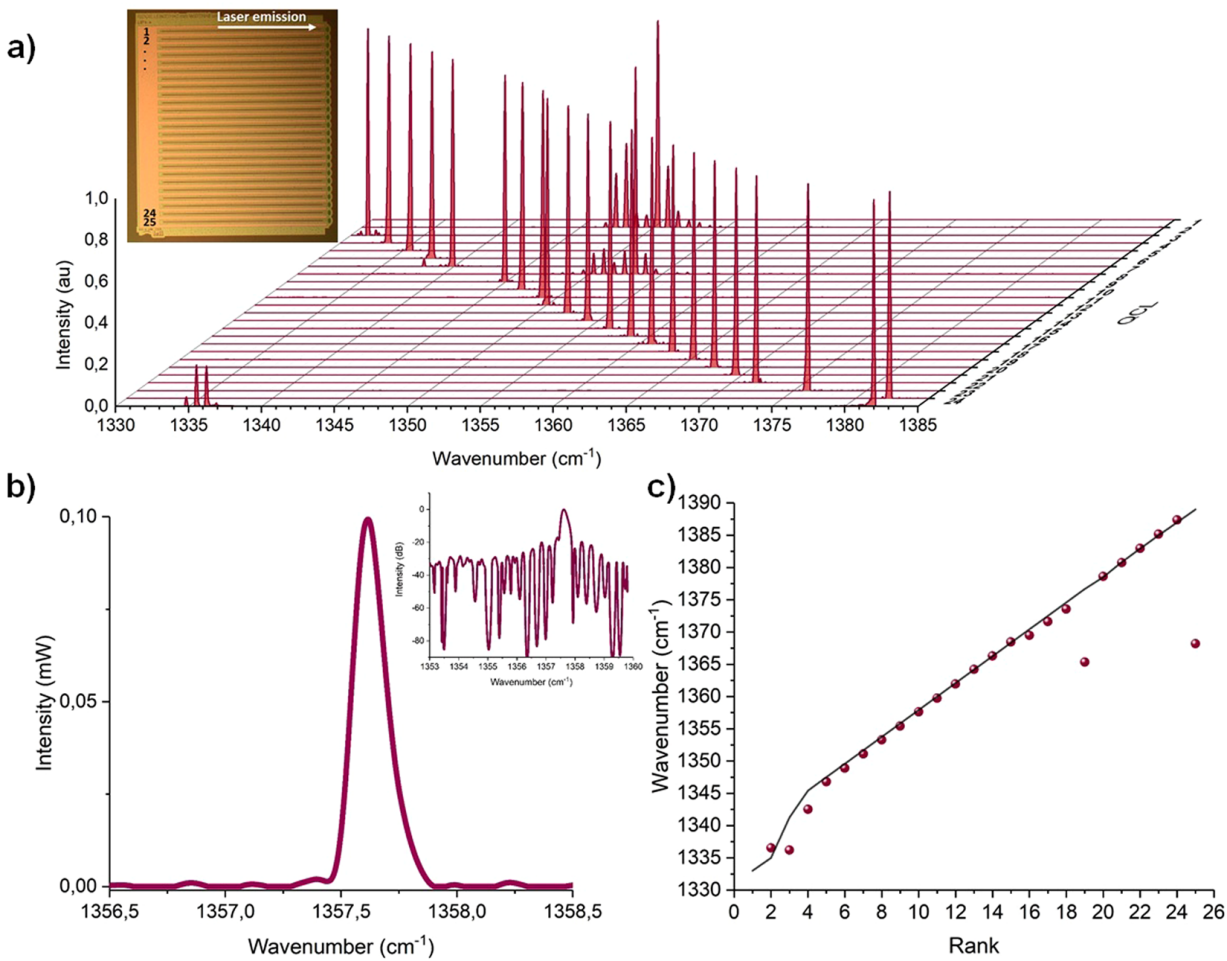
(**a**) Typical spectral power densities of a QCL array (inset: photography of the QCL array, top view) – (**b**) Intensity of a QCL (QCL in mid array; inset: Log representation) – (**c**) Comparison between expected nominal wavenumber and experimental ones.

We also report the normalized typical emission spectra of an array of QCLs working in the 7.4 µm wavelength range for the nominal geometry in Fig. 4a). The output power was measured while operating lasers at the same conditions: applied voltage = 9.9 V, pulsed mode with a 50 ns-pulse duration and 100 kHz-repetition rate (*i.e* 0.5%-duty cycle), operating temperature T=21 °C. The spectral resolution of our Fourier-transform infrared spectrometer (Thermofisher Scientific Nicolet IS-50) is 0.0125 cm^−1^ (4375 points over the spectral range). Apart from sources 2 and 6, the devices showed single-mode emission and 25 dB side mode suppression ratios over the working wavelength range (see Fig. 4b) insert). The emission wavenumbers extracted from this measurement are in good agreement with the expected wavelengths defined by the DFB design, as shown in Fig. 4b). In the Fig. 4c), we plot the intensity emitted by a QCL at the middle of the array (*i.e*. QCL12) with the wavenumber. The maximum power density is 199 µW at 1357.6 cm^−1^. The optical power integrated over the entire emission spectrum corresponds to 18 µW (@0.5% duty cycle). This value is coherent with the power measured in Fig. 3 at 10 V at 1.5%-duty cycle (i.e. 54 µW nearby the threshold). In this operating condition, the typical *FWHM* is 0.16 cm^−1^ with a current pulse duration of 50 ns and a repetition rate at 100 kHz. The *FWHM* is in particular affected by self-heating phenomenon that broadens the linewidth (*FWHM* of a free running QCL is between 1MHz-10MHz in CW mode with an efficient thermal drain)^23^. Other technical noises of the set-up results also in a spectral broadening, and the self-heating contribution cannot be properly quantified with our current set-up. The emission spectrum even widens rapidly for pulses exceeding 100 ns up to 1 cm^−1^ (*FWHM*) for 100ns-pulse duration and 13 V applied voltage.

## Conclusion

In this paper, we report the fabrication of QCL sources on silicon substrate within 200 mm CMOS/MEMS pilot line. To do so, we have successfully transferred the top metal grating process on an appropriate fabrication process flow that fully respects the design and the process rules of a standard CMOS manufacturing line. This fabrication run achieves performance at the state of the art, that are comparable with those of QCL fabricated on InP substrate. The first characterizations done at wafer level have demonstrated average threshold current densities between 2.5 kA/cm² and 3 kA/cm² with a relative dispersion around 3%. The measurements have demonstrated fabrication yields of 98% per wafer having electro-optical features shown in this paper. The optical power can reach 1 mW at ambient temperature, 1.5% duty cycle. This value can easily be increased up to ten mW, which is enough to address many applications in industrial process monitoring or even medical and health fields, by increasing duty cycle value up to a reasonable figure around 15%. Above this repetition rate, the self-heating will probably affect the quantum wells stack and the electro-optical properties will be degraded. Immediate works on this approach will hence consist of studying the self-heating processes and propose a technological way to define a thermal drain toward the silicon tank. In next fabrication batches, up to four 2 inch wafers will be bonded on 200 mm Si wafers for multiplying the number of functional dies available. In parallel, development of InAs/AlSb layers grown by molecular beam epitaxy (MBE) on (100 mm and then 200 mm) silicon substrate^24^ opens new opportunities that should be further explored for a full integration into a CMOS line.

The fabrication in a CMOS/MEMS pilot line and wafer-level tests on probe stations should greatly accelerate the commercialization of QCLs by decreasing the fabrication cost of such components. Furthermore, the integration on a common technological platform implemented on Si substrate is crucial to the realization of miniaturized and cost-effective MIR photonic devices. MIR sources fabricated on Si should penetrate a number of new markets beyond the gas sensing for professional applications.

With these preliminary results, we reached a significant milestone within our works initiated a couple of years ago on passive MIR photonics circuits and integrated photoacoustic detectors^25^. The integration of MIR sources on common technological platforms based on IC/MEMS technology is essential for the realization of miniaturized and cost-effective MIR optical sensors and paving the way for the implementation of a spectrometer fully integrated on Si chip.
